# Fusion genes in cancers: Biogenesis, functions, and therapeutic implications

**DOI:** 10.1016/j.gendis.2025.101536

**Published:** 2025-01-20

**Authors:** Haiqiong Tang, Qiu Peng, Linda Oyang, Shiming Tan, Xianjie Jiang, Zongyao Ren, Xuemeng Xu, Mengzhou Shen, Haofan Li, Mingjing Peng, Longzheng Xia, Wenjuan Yang, Shizhen Li, Jiewen Wang, Yaqian Han, Nayiyuan Wu, Yanyan Tang, Jinguan Lin, Qianjin Liao, Yujuan Zhou

**Affiliations:** a The Affiliated Cancer Hospital of Xiangya School of Medicine, Central South University/Hunan Cancer Hospital, Hunan Key Laboratory of Cancer Metabolism Changsha, Hunan 410013, China; b University of South China, Hengyang, Hunan 421001, China; c Hunan Engineering Research Center of Tumor Organoid Technology and Application, Public Service Platform of Tumor Organoids Technology, Changsha, Hunan 410013, China; d Department of Oncology, Hunan Provincial People's Hospital (The First-Affiliated Hospital of Hunan Normal University), Changsha, Hunan 410005, China

**Keywords:** BCR-ABL, Cancers, EML4-ALK, Fusion genes, PML-RARα

## Abstract

The processes of tumorigenesis and development are intricate, involving numerous genes and molecular pathways. Fusion genes, direct products of abnormal chromosomal rearrangements, are key factors in the formation of many types of tumors. In recent years, the advent of sequencing technology and bioinformatics has led to the discovery of more fusion genes associated with specific types of tumors. The objective of this review is to undertake a comprehensive examination of the discovery and functional mechanisms of fusion genes present in a range of cancers. This will include an analysis of their impact on the biological properties of tumor cells. This review will examine the most prevalent types of fusion genes observed in representative tumor types, including hematological tumors, lung cancer, soft tissue sarcomas, thyroid cancer, and prostate cancer. We provide an overview of the roles and clinical significance of fusion genes, as well as a summarization of the therapeutic strategies for fusion genes, including the application of targeted drugs and related studies. This review presents a comprehensive analysis of the function of fusion genes in the development and treatment of tumors, providing guidance and insights for future research and clinical practice.

## Introduction

In recent years, fusion genes have emerged as a significant area of interest in the field of tumor biology. This is due to the important biological functions they perform as well as the crucial role they play in tumor formation (Table 1). The formation of fusion genes results from aberrant recombination of chromosomal structures, whereby two or more genes that were originally not adjacent are spliced together to generate new genes and proteins. This process often gives rise to the aberrant activation or deactivation of intracellular signaling pathways, which is a significant contributing factor in the development of cancer.^1^ The continuous advancement of molecular biology and genomics technologies has led to the identification of an increasing number of fusion genes. These genes have been extensively studied for their oncogenic mechanisms and applications in tumor diagnosis and therapy.^2^

**Table 1:** The role of fusion genes in the pathogenesis of disease.

Gene fusion	Disease	Function	Reference
EML4-ALK	Non-small-cell lung cancer	The fusion protein sustains activated tyrosine kinase activity and activates multiple signaling pathways involved in the regulation of cell proliferation, survival, migration, and differentiation	20,30,97
ROS1	Non-small-cell lung cancer		
BCL-ABL	Chronic myeloid leukemia		
NPM-ALK	Anaplastic large-cell lymphoma		
ETV6-NTRK3	Congenital fibrosarcoma		
RET-PTC	Thyroid cancer		
PML – RARα	Acute promyelocytic leukemia	It impairs the differentiation of normal cells and stimulates the proliferation of leukemic cells	42,47
AML1-ETO	Acute myeloid leukemia		
SS18-SSX	Synovial sarcoma	The fusion proteins are aberrant transcription factors that interfere with different signaling pathways and target genes, affecting cell proliferation, differentiation, and death	67,106
EWSR1-FLI1	Ewing sarcoma		
ETV6-RUNX1	Acute lymphocytic leukemia	The fusion protein promotes disease by disrupting hematopoietic differentiation, activating proliferative pathways, inhibiting apoptosis, and inducing genomic instability	48,49,52
PAX-FOXO1	Rhabdomyosarcoma	The fusion protein directly affects myoblast differentiation while activating multiple signaling pathways to maintain tumor cell proliferation	75
TMPRSS2-ERG	Prostate cancer	This gene fusion leads to ERG overexpression in response to androgens, which promotes cancer cell proliferation, decreases genomic stability, and changes the tumor microenvironment	101,102

It is well established that fusion genes are a common feature of hematologic and solid tumors, including hematological tumors, lung cancer, soft tissue sarcomas, thyroid cancer, and prostate cancer. The expression products of fusion genes in these tumors have been demonstrated to promote tumor cell proliferation, migration, and survival, and may also affect treatment response.^3^ The development of fusion genes has the potential to provide innovative molecular markers for cancer diagnosis and classification. Additionally, these genes offer new avenues for therapeutic intervention.

However, the field of fusion gene research still faces significant challenges. One of the main hurdles is identifying the diverse functions of these genes. Additionally, there is a need to understand the direct correlation between specific fusion events and malignant transformation. Moreover, the development and refinement of detection techniques with enhanced sensitivity and specificity are crucial in enabling the early and precise identification of fusion genes in patients.^4^ In addition, the development of novel therapies targeting fusion genes is equally challenging, and several obstacles must be overcome. Firstly, it is essential to comprehend how fusion genes influence signaling pathways within diverse cellular microenvironments. Secondly, it is crucial to address the expression heterogeneity of fusion genes in various tumors and across different individuals, including within distinct cancer cells of a single patient. Thirdly, the emergence of resistance to fusion gene-targeted treatments is a significant challenge that necessitates the discovery of effective strategies to overcome such resistance.^5^

The objective of this review is to summarize the latest research on fusion genes and their relationship with various tumors. The aim is to provide a comprehensive understanding of this strong correlation, as well as to delve into the mechanisms by which fusion genes operate and their clinical applications in tumor biology. The review will offer novel ideas and methods for tumor research and treatment.

## Characterization of fusion genes

**Figure 1:**
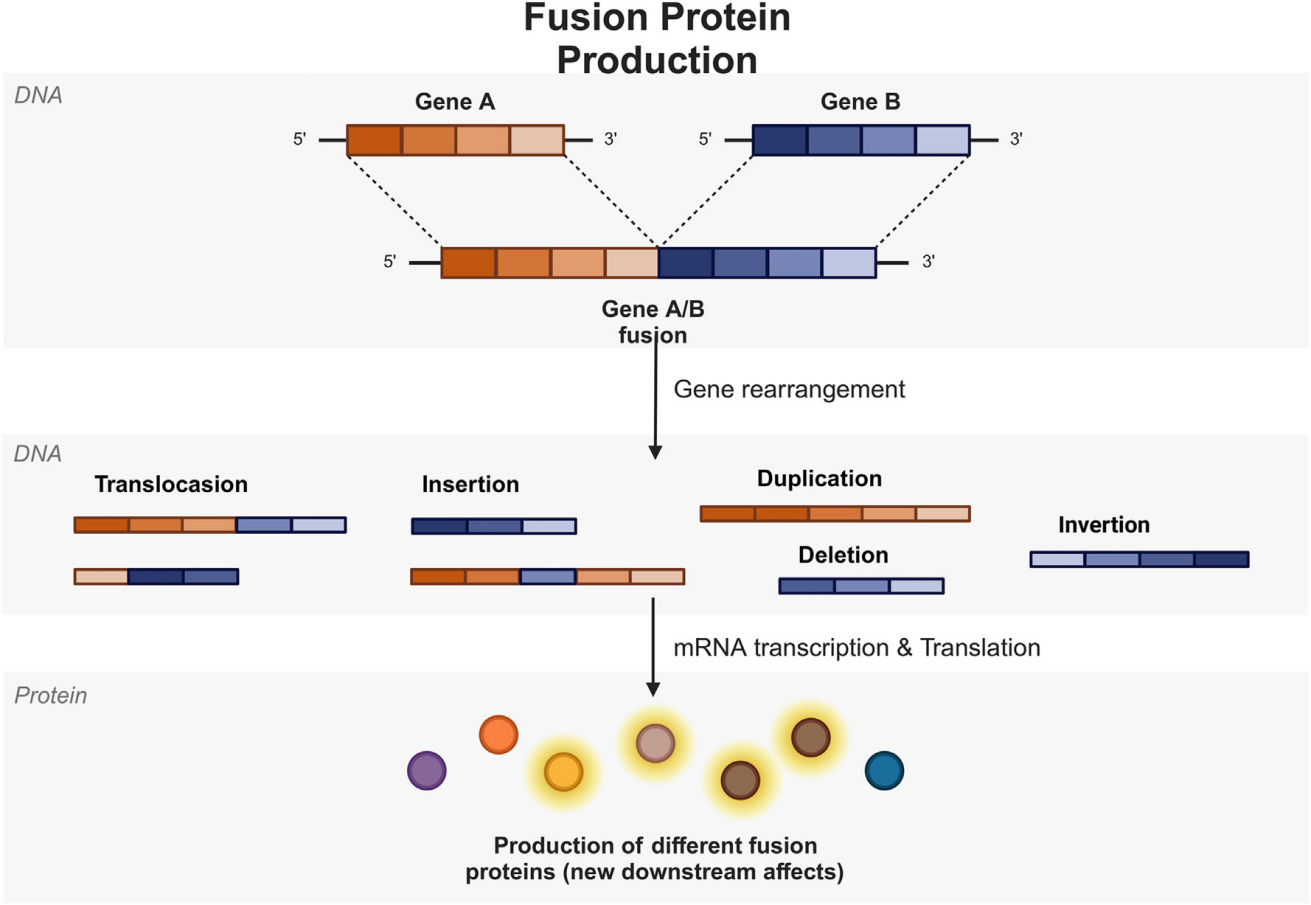
Formation of fusion proteins. New genes are formed of two originally independent genes by chromosomal rearrangements, including translocations, insertions, deletions, duplications, and inversions. These new genes are transcribed and translated to form different fusion proteins that affect downstream signaling pathways.

A fusion gene is defined as one that arises through the aberrant combination of two previously distinct genes. Such recombination events can be complex, involving the translocation, insertion, duplication, deletion, inversion, or other complex rearrangements of chromosomes (Fig. 1).^1^ The rapid development of detection technologies has prompted a detailed investigation into fusion genes, revealing several characteristics. Disease diagnosis is a prominent area of research, with certain fusion genes exhibiting high associations with specific diseases. These genes may serve as diagnostic biomarkers. For example, the EML4 (echinoderm microtubule-associated protein-like 4)-ALK (anaplastic lymphoma kinase) fusion gene has been linked to non-small cell lung cancer, while BCR-ABL has been associated with chronic myeloid leukemia. The products of fusion genes may have the ability to activate cellular proliferation, thereby promoting tumor development. Due to their unique nature and critical role in tumors, fusion genes and their products are targets for targeted therapy. In some tumors, the presence of fusion genes can provide insight into the disease course and patient prognosis.^2^^,^^6^, ^7^, ^8^

## The testing technologies of fusion genes

From the beginning, fusion gene detection techniques have been divided into two main types: traditional methods and new technologies. Traditional methods include fluorescence *in situ* hybridization, reverse transcription PCR, and immunohistochemistry. While these methods are very sensitive, they have limitations in identifying new fusion genes or proteins.^9^, ^10^, ^11^ Conversely, the predominant detection methodology employed is high-throughput detection technology, including RNA sequencing and DNA sequencing. This technology is adept at identifying previously unrecognized fusion events; nevertheless, the data analysis process is intrinsically complex and costly.^12^^,^^13^ Emerging diagnostic technologies, such as liquid biopsy, mass spectrometry, and nanopore sequencing, are advancing swiftly and are increasingly utilized for the identification of fusion genes. Liquid biopsy, in particular, enables the detection of fusion gene sequences within circulating tumor DNA or RNA. The primary advantages of this technology include its simplicity, non-invasiveness, and suitability for the dynamic monitoring of tumor progression.^14^ Mass spectrometry offers significant advantages in fusion genetic testing compared with DNA and RNA analyses, as it provides insights at the proteomic level. Nevertheless, the application of mass spectrometry necessitates high-quality samples and involves intricate bioinformatics analysis, rendering the process both time-intensive and expensive.^15^ Nanopore sequencing possesses the capability to produce long reads, enable swift analysis, and identify previously unrecognized fusion genes. Nanopore sequencing demonstrates high sensitivity and specificity in detecting fusion genes, with the ability to identify fusion gene events at frequencies as low as 1%. However, it is important to recognize the existing limitations of the analytical tools used in this context.^16^^,^^17^ The future evolution of fusion genetic testing technology will be contingent upon the advancement of four fundamental principles: high sensitivity, portability, dynamic monitoring, and multi-omics integration. These advancements aim to propel the progress of precision medicine and personalized treatment strategies. The incorporation of emerging technologies and artificial intelligence is expected to augment the capabilities of fusion genetic testing, thereby enabling more effective early diagnosis, treatment selection, and prognosis assessment in oncology.

## Roles of fusion genes in lung cancer

Lung cancer is one of the most prevalent forms of cancer worldwide and is consistently ranked as the leading cause of cancer-related deaths globally. In 2022, lung cancer accounted for 22% of all malignant tumor incidences and 28.5% of all malignant tumor deaths in China, making it the most prevalent and deadly form of cancer in the country.^18^ Lung cancer can be divided into two major groups: small-cell lung cancer and non-small-cell lung cancer. The latter accounts for approximately 85% of cases. In recent years, researchers have identified the ALK-ROS1 (Ros proto-oncogene 1) fusion gene and confirmed both genes as driver genes that promote the progression of lung cancer. Targeted drugs have been developed against these two targets, and they have shown excellent therapeutic efficacy in patients who are positive for the driver genes.^19^

**Figure 2:**
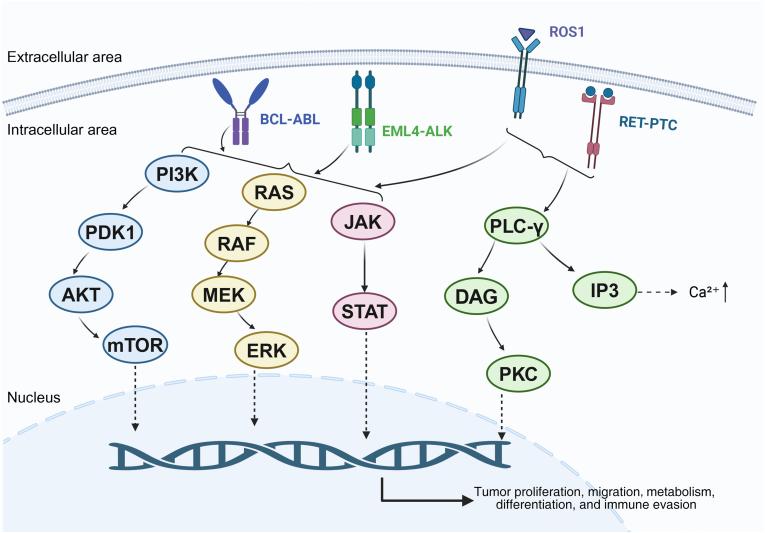
Tumor signaling pathways regulated by fusion genes. BCL-ABL fusion proteins and EML4-ALK fusion proteins are present intracellularly, and both fusion proteins have sustained activated tyrosine kinase activity. This abnormally activated tyrosine kinase is capable of sequentially activating multiple downstream signaling pathways, including PI3K/PDK1/AKT/mTOR, RAS/RAF/MEK/ERK, and JAK/STAT. ROS1 fusion proteins are typically located on the cell membrane. ROS1 is a tyrosine kinase receptor. When partially fused to other proteins, it can result in sustained activity of its kinase, which in turn activates multiple intracellular signaling pathways, including PI3K/PDK1/AKT/mTOR, RAS/RAF/MEK/ERK, JAK/STAT, and PLC-γ/DAG/PKC. The RET-PTC fusion protein is located intracellularly, and the generation of fusion events leads to the ability of RET kinase to activate in the absence of a ligand. This sustained activation of RET kinase activates multiple intracellular signaling pathways, including PI3K/PDK1/AKT/mTOR, RAS/RAF/MEK/ERK, JAK/STAT, and PLC-γ/DAG/PKC.

The EML4-ALK fusion gene was initially identified by Japanese researchers in the tumor tissue of a male adenocarcinoma patient with a history of smoking. This fusion gene produces a fusion protein with kinase activity that activates multiple signaling pathways, including JAK (Janus kinase)/STAT (signal transducer and activator of transcription), PI3K (phosphoinositide 3-kinase)/AKT (protein kinase B), and RAS/MAPK (mitogen-activated protein kinase), to promote tumor cell growth and survival (Fig. 2). This aberrant kinase activity results in increased cell proliferation and sustained activation of signaling pathways, ultimately driving tumor progression.^20^ Inhibitors targeting EML4-ALK fusion proteins have emerged as crucial therapeutics for ALK-positive non-small cell lung cancer. However, recent research^21^ suggests that ALK fusion oncogenes may enhance tumor cell viability by up-regulating SERPINB4 (serpin family B member 4) expression and inhibiting the immunoactivity of natural killer cells, enabling tumor cells to evade immune clearance. This finding provides valuable insights for further investigation into the immune evasion mechanism of ALK-fusion non-small-cell lung cancer, as well as novel approaches for developing therapeutic strategies targeting this oncogenic pathway. The EML4-ALK fusion gene leads to the aberrant activation of ALK kinase, thereby facilitating the growth and proliferation of tumor cells. In 2011, the first tyrosine kinase inhibitor (TKI) targeting ALK, crizotinib, was approved by the US Food and Drug Administration. It works by inhibiting the activity of this abnormal kinase, preventing tumor cells from growing and metastasizing. As a result, crizotinib is the first-line treatment for patients who are positive for the EML4-ALK fusion gene. However, studies have shown that some patients develop drug resistance to crizotinib after approximately 12 months of treatment.^22^ Secondary mutations, gene amplification, and phenotypic switching are among the causes of drug resistance. Secondary mutations can lead to changes in the original binding sites of ALK-TKIs. This can reduce the drug's affinity for tumor cells and diminish its efficacy. Tumor cells may develop resistance to the drug by up-regulating the copy number of the ALK gene, thereby counteracting its inhibitory effect. Another contributing factor is phenotypic switching, where tumor cells may undergo a transformation into small-cell lung cancer with more aggressive features or exhibit epithelial–mesenchymal transition features, leading to the ineffectiveness of ALK-TKIs. Additionally, other pathways of the kinase signaling pathway may allow tumor cells to evade the inhibitory effects of ALK-TKIs. Tumor cells may develop drug resistance through various mechanisms, including overexpressing certain proteins (*e.g.*, epidermal growth factor receptor/EGFR, insulin-like growth factor-1 receptor/IGF-1R, c-KIT), which can lead to inhibition of drug efficacy. Tumor cells can also up-regulate the expression of certain drug pumps, such as P-glycoprotein, which can pump drugs out of the cell and reduce the internal drug levels, thereby reducing the effectiveness of treatment.^23^^,^^24^ To overcome ALK mutations associated with drug resistance, researchers have developed second-generation and third-generation ALK-TKIs, such as ceritinib and loratinib. However, alternative approaches or novel ALK inhibitors should be explored as long-term use of ALK-TKIs may lead to drug resistance. Hong et al^25^ demonstrated that the EML4-ALK fusion protein markedly up-regulates the expression of programmed death-ligand 1 (PD-L1) by activating downstream signaling pathways, including STAT3 and PI3K/AKT. Furthermore, the study observed that increased PD-L1 expression impaired effector T cell functionality and enhanced the immune evasion capabilities of tumors in a murine model. In contrast, administration of anti-PD-1 (programmed death-1)/PD-L1 immunotherapy restored the anti-tumor activity of T cells and significantly reduced tumor burden. These findings suggest the potential of combining ALK-TKIs with anti-PD-1/PD-L1 therapy, especially in patients who have developed resistance to other treatments, as immune checkpoint inhibitors like PD-1/PD-L1 inhibitors may provide a novel therapeutic approach. Gao et al^26^ synthesized a new ALK inhibitor called ZYY-B-2, which blocks the growth of tumor cells by inactivating downstream target proteins. Notably, ZYY-B-2 inhibits this process by blocking the function of P-gp, a protein that excretes drugs out of cells, leading to decreased drug concentration and drug resistance. Inhibiting ZYY-B-2 can prevent this process, allowing ceritinib to maintain a certain intracellular concentration and overcome resistance in ceritinib-resistant H2228 cells. Additionally, ZYY-B-2 can induce apoptosis in H2228 cells through the mitochondrial pathway, which involves lowering the mitochondrial membrane potential, releasing cytochrome c into the cytoplasm, and activating pro-apoptotic caspases. The study found that ZYY-B-2 could not only inhibit the growth of cancer cells but also promote the death of the cancer cells. This study indicates that ZYY-B-2 may be a potential therapeutic to overcome ALK rearrangement-positive lung cancer cell resistance to ceritinib. Another extensively researched therapeutic strategy is the inhibition of HSP90. HSP90 is a molecular chaperone protein that regulates the folding, stability, and function of other proteins. Cancer cells are often highly dependent on HSP90 for survival, with ALK fusion proteins being particularly sensitive to HSP90 inhibitors.^27^ These inhibitors bind to HSP90 proteins, preventing them from binding to oncogenic proteins and resulting in the degradation or inactivation of these proteins. Some of these inhibitors have shown promising therapeutic effects in clinical trials.^23^ HSP90 inhibitors face several challenges in clinical practice despite their promising applications. Toxic side effects such as liver toxicity, visual disturbances, and gastrointestinal problems must be managed. Additionally, individual patients may develop resistance to HSP90 inhibitors. Further studies and clinical practice are necessary for validation of their efficacy and safety, and as a result, they have not yet been widely used in clinical practice. Further studies are needed to determine whether the combination of HSP90 inhibitors with ALK-TKIs will improve efficacy and reduce side effects in patients.

ROS1 is a type of rearrangement that is found in non-small cell lung cancer. ROS1 gene fuses with another gene at the chromosomal level to form a new fusion gene. Although only 1%–2% of lung cancer patients are found to carry the ROS1 fusion gene, there is still a significant number of patients with the disease. The ROS1 fusion gene is more prevalent in younger women with no history of smoking and adenocarcinoma pathology.^28^^,^^29^ This fusion gene has the potential to activate cellular signaling pathways such as PI3K/AKT, MAPK, and JAK/STAT, which may contribute to cancer cell proliferation, survival, and metastasis.^30^ In the treatment of non-small-cell lung cancer, inhibitors targeting the ROS1 fusion gene have become a major focus of research. Tumor growth and metastasis can be inhibited by blocking these aberrantly activated signaling pathways. ROS1 protein shares significant homology with ALK, particularly in the ATP-binding site and the kinase structural domain 31. Crizotinib is an ALK kinase inhibitor. It is as effective against the ROS1 fusion gene as it is against ALK fusion genes. However, long-term use of kinase inhibitors can be a source of drug resistance. Lyer et al^32^ discovered that the expression level of the MYC gene was significantly up-regulated in ROS1 fusion-positive lung cancer cells after long-term use of TKIs. This up-regulation was strongly correlated with the development of drug resistance to TKIs. MYC up-regulation may promote tumor cell survival and reduce the toxic effects of TKIs by regulating cell metabolism and reducing apoptosis. Inhibiting MYC activity has been shown to enhance the efficacy of TKIs. Investigators established patient-derived xenografts of LUAD-0006 with MYC amplification. After down-regulating MYC with docetaxel, they found that co-treatment of LUAD-0006 cells with crizotinib and MYC knockdown completely inhibited cell proliferation. Additionally, robust overexpression of MYC in LUAD-0006 cells resulted in a significant loss of efficacy of the ROS1 kinase inhibitor. To address this issue, the researchers selected CDK4/6 (cyclin-dependent kinase 4) inhibitors and BET bromodomain inhibitors to inhibit LUAD-0006 cell viability. *In vitro* data further confirmed that these two inhibitors could work synergistically with crizotinib to resensitize MYC-overexpressing ROS1 cells to crizotinib. This offers more opportunities for patients with resistance to existing ROS1 kinase inhibitors. Similarly, Liu et al have opened up a new therapeutic avenue for patients who are resistant to ROS1 kinase inhibitors. Liu et al^33^ synthesized a novel class of 2-aminopyridine derivatives acting as dual inhibitors of ROS1 and ALK. These derivatives were shown to have potent inhibitory effects in *ex vivo* and *in vivo* experimental models. The authors also predicted the binding modes of the 2-aminopyridine derivatives to the ROS1 and ALK enzymes, further supporting their use as dual inhibitors. Liu et al investigated the C01 compounds, which exhibit anti-proliferative activity against ROS1 and ALK, including drug-resistant mutants such as ROS1G2032R and ALKG1202R. This discovery provides a new therapeutic option for the treatment of patients with ROS1- or ALK-positive lung cancer. As research progresses, more ideas for treatment are emerging.

## Roles of fusion genes in hematological tumors

In the 1960s, a specific translocation known as the t(9; 22) (q34; q11) chromosome translocation, also referred to as the Philadelphia chromosome translocation, was observed in the chromosomes of patients with chronic myeloid leukemia. This translocation is caused by the translocation of the leukemia proto-oncogene ABL located on the distal end of the long arm of chromosome 9 to the BCR gene located on chromosome 22.^34^, ^35^, ^36^ The heterogeneous breakpoints within the BCR and ABL1 genes result in variations in their respective molecular weights and pathogenic mechanisms, consequently leading to distinct disease phenotypes. This phenomenon gives rise to three primary BCR-ABL1 fusion proteins of differing sizes. One of these is the p190 BCR-ABL1, encoded by the e1a2 fusion transcript, which has a molecular weight of 190 kDa. The p190 variant is predominantly associated with acute lymphoblastic leukemia, although it can also be detected in a limited number of chronic myeloid leukemia cases. The p230 BCR-ABL1 protein is encoded by either a b2a3 or b3a3 fusion transcript and has a molecular weight of 230 kDa. Although primarily associated with chronic myeloid leukemia, p230 BCR-ABL1 is also linked to a rare variant of chronic neutrophilic leukemia or a slower-progressing form of chronic myeloid leukemia. This variant is characterized by milder clinical symptoms and a more gradual disease progression. The p210 BCR-ABL1 protein, encoded by either a b2a2 or b3a2 fusion transcript and possessing a molecular weight of 210 kDa, represents the prototypical fusion protein associated with chronic myeloid leukemia and serves as the molecular hallmark in the majority of chronic myeloid leukemia cases. This protein exhibits abnormal tyrosine kinase activity, leading to the dysregulation of cellular proliferation and survival, thus facilitating the advancement of leukemia.^34^^,^^37^^,^^38^ Feng et al^39^ found that the expression level of PHF8 (PHD finger protein 8) was significantly elevated in chronic myeloid leukemia and was associated with a poor prognosis of the patients. The results suggest that PHF8 promotes the development of chronic myeloid leukemia by directly regulating the expression of the BCR-ABL1 gene through demethylation. Inhibition of PHF8 activity was found to reduce BCR-ABL1 expression and promote differentiation and inhibit proliferation of chronic myeloid leukemia cells. Before the application of TKIs in the treatment of chronic myeloid leukemia, therapies mainly consisted of interferon-alpha therapy, palliative chemotherapy, radiotherapy, and hematopoietic stem cell transplantation. However, a new era of targeted therapy for chronic myeloid leukemia began with the discovery of the BCL-ABL fusion gene. This led to the development of imatinib. Imatinib is a TKI that targets the activity of BCL-ABL kinase, thereby inhibiting tumor growth without affecting the cells of the normal tissue. Patients with chronic myeloid leukemia who were resistant or intolerant to interferon-alpha were studied in a phase I clinical trial of imatinib. Up to 98% of the patients achieved complete hematologic remission and 13% achieved complete cytogenetic remission. In a phase III clinical trial in patients with first-diagnosed and untreated chronic myeloid leukemia, the control group received treatment with interferon-alpha and cytarabine, while the study group received treatment with imatinib. The complete cytogenetic remission rate was more than eight times higher in the imatinib group than in the interferon-alpha plus cytarabine group.^40^ Imatinib has become the primary treatment for chronic myeloid leukemia and has significantly prolonged patient survival. However, long-term use of the drug can lead to the emergence of BCL-ABL kinase structural domain mutations and acquired resistance. Although researchers have explored a new generation of TKIs, it is still a challenge to avoid the toxic side effects of the drugs. Therefore, new ideas and treatments need to be discovered. Gao et al^41^ investigated a drug named I13, which directly inhibited the BCL-ABL protein, overcoming the resistance caused by the T315I mutation in chronic myeloid leukemia. The studies indicate that I13 can effectively inhibit the BCR-ABL protein with the T315I mutation. It blocks the proliferation of and induces the differentiation of chronic myeloid leukemia cells with T315I mutation and wild-type BCR-ABL, as observed in colony formation assays. These findings suggest that I13 may be a promising therapeutic agent. This discovery has significant implications for the development of new therapeutic agents targeting BCR-ABL1.

Similar pathogenic fusion genes are found in leukemias, such as AML1 (acute myeloid leukemia-1)-ETO (eight twenty-one), PML (promyelocytic leukemia)-RARα (retinoic acid receptor alpha), and ETV6 (ETS variant transcription factor 6)-RUNX1 (RUNX family transcription factor 1). The latter is a marker for acute promyelocytic leukemia (APL) and is present in over 90% of APL patients. PML-RARα is caused by a balanced translocation between the PML gene on chromosome 15 and the RARA gene on chromosome 17. The protein encoded by the fusion gene disrupts cell differentiation, cell cycle regulation, and apoptosis, leading to the development of APL.^42^ The identification of the PML-RARα fusion gene has not only provided an understanding of the molecular etiology of APL but also revealed its therapeutic target. The therapeutic potential of all-trans retinoic acid for leukemia was discovered by researchers in the 1970s. Specifically, it was found to be highly effective in APL patients, causing leukemia cells to differentiate and mature, resulting in therapeutic efficacy.^43^ However, the long-term use of all-trans retinoic acid led to the development of drug resistance. The use of arsenic trioxide, which induces apoptosis, was used to overcome this problem. The results showed that the combination of the two drugs achieved favorable results compared with all-trans retinoic acid alone, with a higher rate of complete remission, a higher rate of PML-RARα conversion, and a higher 2-year survival rate, as well as a lower rate of side effects and relapse, than the treatment with all-trans retinoic acid alone. APL cure rates have increased dramatically with the combination of all-trans retinoic acid and arsenic trioxide. New therapeutic opportunities for the treatment of ATRA-resistant APL have been identified in recent studies. Shao et al^44^ proposed a new way for the treatment of APL and drug-resistant APL by destabilizing PML-RARα. Deubiquitinating enzymes (DUB) specifically unfold the ubiquitin chain to the substrate to achieve the maintenance of the stability of PML-RARα protein, making DUB a new target for anti-tumor therapy. Using DUB siRNA libraries, they identified the deubiquitinating enzyme YOD1 as a key DUB for PML-RARα. They found that inhibiting YOD1 leads to deubiquitination and degradation of PML-RARα. This result was further confirmed by experiments that blocked YOD1 from degrading oncogenic PML-RARα and eliminated APL cells.

AML1-ETO is a fusion gene that results from the translocation of the ETO gene located on chromosome 8 with the AML1 gene located on chromosome 22. This genetic abnormality is commonly found in acute myeloid leukemia and can disrupt the development and function of normal hematopoietic cells. Additionally, research has demonstrated that the AML1-ETO fusion gene can interact with other signaling pathways that further affect cell proliferation and survival, ultimately leading to the development of acute myeloid leukemia.^45^^,^^46^ A recent study by Golovine et al^47^ has highlighted the significant role of ABL1 kinase in AML1-ETO and NUP98 (nucleoporin 98)-PMX1 (paired related homeobox 1) leukemias. They discovered that the down-regulation of ABL1 kinase activity in AML1-ETO and NUP98-PMX1 leukemias is associated with leukemia initiation and progression. Using mouse models and human leukemia cell lines, the scientists demonstrated that ABL1 kinase may act as a tumor suppressor in these cancers by regulating cell proliferation, apoptosis, and differentiation. Furthermore, the researchers discovered that activating ABL1 kinase may alter the sensitivity of these two fusion genes to DNA damage response inhibitors, which could impede leukemia progression. This indicates that ABL1 kinase may be a therapeutic target. Currently, chemotherapy and hematopoietic stem cell transplantation are the mainstay of treatment for acute myeloid leukemia caused by the AML1-ETO fusion gene. Targeted therapies for the AML1-ETO fusion gene are currently under investigation. These include targeted drugs for proteins produced by the fusion gene and other novel therapeutic approaches. It is expected that better drugs will become available in the future to improve the prognosis of acute myeloid leukemia patients.

The ETV6-RUNX1 fusion gene is a common genetic anomaly in acute lymphoblastic leukemia, especially prevalent in pediatric cases of B-cell acute lymphoblastic leukemia, where it is detected in approximately 25% of patients. This genetic alteration arises from a chromosomal translocation involving the ETV6 and RUNX1 genes, typically presenting as the t(12; 21) (p13; q22) translocation between chromosomes 12 and 21.^48^^,^^49^ According to the findings of Jeha et al^48^ and Sundaresh et al,^50^ patients exhibiting ETV6-RUNX1 positivity demonstrate significantly increased sensitivity to chemotherapy, achieving a five-year event-free survival rate of 95%. As a result, the presence of the ETV6-RUNX1 fusion gene is considered indicative of a favorable prognosis. Furthermore, immunotherapy has emerged as a subject of substantial interest in the treatment of B-cell acute lymphoblastic leukemia. A clinical investigation by Maude et al^51^ has established that tisagenlecleucel provides a more effective and targeted therapeutic option compared with conventional treatments, such as chemotherapy and hematopoietic stem cell transplantation, for patients with relapsed or refractory B-cell acute lymphoblastic leukemia. Tisagenlecleucel is the first treatment to receive approval for use in pediatric and young adult patients with relapsed/refractory B-cell acute lymphoblastic leukemia, representing a pivotal advancement in the application of chimeric antigen receptor T-cell therapy for hematological malignancies. Despite extensive research efforts, the pathogenic mechanism of the ETV6-RUNX1 fusion gene remains only partially elucidated. It is well-established that the fusion gene, in isolation, does not typically culminate in the onset of leukemia. Instead, it is considered an “initiating mutation”, signifying its critical role in the early stages of leukemogenesis, necessitating the presence of additional mutations for the full development of leukemia. Secondary mutations in other genes (paired box 5/PAX5, cyclin-dependent kinase inhibitor 2A/CDKN2A, *etc*.) are postulated to be requisite for the full development of leukemia, and together they accelerate the onset and progression of leukemia.^52^ Rodríguez-Hernández et al^53^ discovered that the ETV6-RUNX1 fusion gene exhibited only partial precancerous characteristics in B-cell precursors, yet insufficient to induce leukemia. Furthermore, they observed that B-cell precursors could only be fully transformed into leukemic cells in the presence of additional genetic alterations, such as RAS mutations, PAX5 deletions, and CDKN2A deletions. The prognosis of patients with various types of secondary oncogenic mutations exhibits significant variability. Mutations that promote proliferation, such as KRAS mutations, are correlated with a poorer prognosis and an increased risk of relapse. In contrast, mutations that impede differentiation, such as PAX5 deletions, have been associated with drug resistance. The findings of this study may contribute to the development of more personalized treatment strategies for patients harboring the ETV6-RUNX1 fusion gene. Furthermore, they could contribute to the prognostic assessment and risk stratification of patients by identifying different types of second oncogenic hits. Sharma et al^54^ validated the synergistic effect of IGF2BP1 (insulin-like growth factor 2 mRNA binding protein 1) with ETV6-RUNX1 and revealed the role of IGF2BP1 through mRNA stabilization in the oncogenic process of B-cell acute lymphoblastic leukemia. The expression level of IGF2BP1 in ETV6-RUNX1-positive B-cell acute lymphoblastic leukemia cells was investigated, revealing a significant up-regulation of this gene in the leukemia cells. Furthermore, knockdown experiments targeting either IGF2BP1 or ETV6-RUNX1 led to a reduction in the proliferative capacity and an increase in apoptosis of the leukemia cells. Additionally, further studies have shown that IGF2BP1 interacts with and stabilizes specific oncogenic mRNAs, thereby promoting their expression. Conversely, the ETV6-RUNX1 fusion gene has been demonstrated to activate downstream pro-oncogenic pathways via transcriptional regulation. This synergistic effect amplifies oncogenic signaling within tumors. In summary, the ETV6-RUNX1 fusion gene constitutes a critical molecular marker for childhood acute lymphoblastic leukemia. Early detection of this gene is essential for the development of effective therapeutic strategies.

Within the spectrum of hematologic malignancies, fusion genes are also observed in non-Hodgkin's lymphomas, which originate within the lymphatic system. A notable example is the NPM (nucleophosmin)-ALK fusion gene, frequently identified in anaplastic large cell lymphoma. This fusion gene typically arises from a chromosomal translocation involving the ALK gene on chromosome 2 and the NPM gene on chromosome 5, culminating in a t(2; 5) (p23; q35) translocation.^55^^,^^56^ The NPM-ALK fusion gene encodes a chimeric protein that integrates the N-terminal segment of the NPM gene with the C-terminal tyrosine kinase domain of the ALK gene. The structural domain of NPM facilitates protein dimerization, which subsequently activates the tyrosine kinase activity of ALK, leading to the persistent initiation of downstream signaling cascades. These activated pathways include JAK/STAT, PI3K/AKT, RAS/MAPK, and NF-κB (nuclear factor kappa B). Consequently, the continuous activation of these pathways renders NPM-ALK a potent oncogenic driver.^56^^,^^57^ The management of non-Hodgkin's lymphoma continues to predominantly rely on chemotherapy, specifically utilizing the CHOP regimen (cyclophosphamide, doxorubicin, vincristine, and prednisone). Nevertheless, for patients with NPM-ALK-positive subtypes, two targeted therapeutic approaches are available: one involves the use of an ALK inhibitor, while the other targets CD30, a surface marker molecule that is highly expressed on anaplastic large-cell lymphoma cells. Similar to the therapeutic outcomes observed in EML4-ALK-positive lung cancer, patients with NPM-ALK-positive lymphomas have shown significant responsiveness to all generations of ALK inhibitors. Notably, agents such as crizotinib, alectinib, and ceritinib have demonstrated considerable efficacy in this patient population. Another targeted therapeutic agent is brentuximab vedotin, which eliminates tumor cells by binding to CD30, a marker molecule abundantly expressed on the surface of anaplastic large-cell lymphoma cells, and subsequently releasing cytotoxic agents.^58^^,^^59^ BRG1 (Brahma-related gene 1), an ATPase core subunit of the SWI/SNF complex, is instrumental in the regulation of chromatin remodeling, thereby influencing the transcriptional activity of genes. This mechanism is particularly significant in the context of various cancers.^60^ Garland et al^61^ demonstrated that elevated BRG1 expression in anaplastic large-cell lymphoma cells is closely associated with the expression of NPM-ALK fusion genes. Moreover, the authors suggested that NPM-ALK might modulate BRG1 expression via signaling pathways such as NF-κB. The suppression of BRG1 expression, achieved through the application of BRG1 inhibitors or siRNAs, led to a reduction in biological activities, including proliferation, survival, and migration, in anaplastic large-cell lymphoma cells. Furthermore, the inhibitory impact of BRG1 suppression on tumor activity was found to be dose-dependent. The study conducted by the team revealed that the inhibition of BRG1 significantly impeded the proliferation and survival of anaplastic large-cell lymphoma cells, suggesting that BRG1 could serve as a viable target for anaplastic large-cell lymphoma treatment. Future development of pharmacological agents targeting BRG1 may offer novel therapeutic strategies for anaplastic large-cell lymphoma patients, especially in scenarios where resistance to chemotherapy presents a significant challenge.

## Roles of fusion genes in soft tissue sarcomas

Among the fusion genes frequently identified in soft tissue tumors are SS18 (synovial sarcoma 18)-SSX (synovial sarcoma X) in synovial sarcoma, EWSR1 (EWS RNA binding protein 1)-FLI1 (friend leukemia virus integration 1) in Ewing sarcoma, PAX (paired box)-FOXO1 (forkhead box O1) in rhabdomyosarcoma, and ETV6-NTRK3 (neurotrophic receptor tyrosine kinase 3) in congenital fibrosarcoma.

Synovial sarcoma is a rare malignant tumor that originates in the synovial tissue of a joint. It typically occurs near large joints, such as the knees, shoulders, and hips, but may occur in other areas as well. This tumor grows rapidly and can invade surrounding tissues and bones, causing joint swelling, pain, limited movement, and dysfunction. Studies have shown that patients with synovial sarcoma frequently exhibit chromosomal abnormalities, usually involving a translocation between chromosomes X and 18, resulting in the formation of several fusion genes, including SS18-SSX1, SS18-SSX2, and SS18-SSX4. The production of these fusion genes is thought to be crucial in the development of synovial sarcoma.^62^^,^^63^ The pathogenic mechanisms of the SS18-SSX fusion genes are still under continuous investigation. One mechanism suggests that the SS18-SSX fusion gene may affect the transcriptional regulatory network, resulting in the abnormal expression of a variety of genes, including those associated with cell proliferation, apoptosis, differentiation, and invasion, and thereby promoting the abnormal proliferation, survival, and metastatic ability of tumor cells. Cyra et al^64^ elucidated the role of the SS18-SSX fusion protein in facilitating CREB (cyclic-AMP response element-binding) activation within synovial sarcoma. The initial observations revealed that the expression of SS18-SSX led to elevated levels of CREB protein and enhanced phosphorylation at the S133 site in SCP-1 mesenchymal stem cells. This function of SS18-SSX was further validated across three synovial sarcoma cell lines, where it was demonstrated that both CREB S133 phosphorylation and the expression of CREB downstream targets were contingent upon the presence of SS18-SSX. The research team conducted additional experiments, which demonstrated that the depletion of CREB led to a significant reduction in the viability of synovial sarcoma cells. Furthermore, they observed that these cells exhibited sensitivity to the CREB inhibitor 666-15. The findings indicated that 666-15 markedly impaired cell viability and induced apoptosis in synovial sarcoma cell lines, as evidenced by both *in vitro* and *in vivo* experiments. These results suggest that the SS18-SSX fusion protein may be vulnerable to the inhibitory effects of 666-15. The presence of SS18-SSX fusion proteins is a hallmark feature of synovial sarcoma, and studies on the mechanism of CREB activation may help to develop new therapeutic approaches against synovial sarcoma. Currently, the treatment of synovial sarcoma patients is based on surgery, with appropriate postoperative radiotherapy and/or chemotherapy. However, existing treatment options for patients with advanced synovial sarcoma are ineffective. Targeted therapy and immunotherapy are currently under clinical investigation, and these new treatments offer hope to patients.

Ewing sarcoma is a rare malignant bone tumor that usually occurs in children and young adults. It commonly develops in long bones, such as the thigh or hip, but may also occur in other areas. Symptoms include localized pain, lumps, and fractures. Ewing sarcoma arises from a chromosomal translocation event involving the EWSR1 and FLI1 genes, which can lead to the generation of the EWSR1-FLI1 fusion gene.^65^^,^^66^ The EWSR1-FLI1 fusion protein is a key factor in the development of Ewing sarcoma, which can promote tumor cell growth and metastasis by regulating the abnormal activation of various cellular signaling pathways.^67^ Additionally, the EWSR1-FLI1 fusion protein interacts with other proteins and affects the regulatory network of gene expression, resulting in the abnormal expression of tumor-related genes. The treatment of Ewing sarcoma usually includes a combination of chemotherapy, surgery, and radiotherapy. However, with the continuous development of gene editing technology. Cervera et al^68^ explored the therapeutic potential of targeting and inactivating the EWSR1-FLI1 fusion gene using CRISPR/Cas9 technology. The researchers employed CRISPR/Cas9 gene editing to design sgRNAs aimed at the breakpoints of the EWSR1-FLI1 fusion gene, with the specific goal of cleaving the fusion gene. Subsequently, both *in vitro* and *in vivo* experiments were conducted to evaluate cell proliferation, survival, and apoptosis following the inactivation of EWSR1-FLI1 in Ewing sarcoma cells. The inactivation of EWSR1-FLI1 was observed to significantly impede tumor growth following the localized administration of the CRISPR/Cas9 system to the tumor site. These results offer compelling evidence supporting the potential of gene editing as a therapeutic strategy for Ewing sarcoma, indicating the feasibility of developing novel precision therapies targeting fusion genes. Seong et al^69^ reported a significantly increased expression of TRIM8 (tripartite motif containing 8) in both Ewing sarcoma cells and patient samples, which demonstrated a positive correlation with the proliferative capacity of the tumor cells. The study demonstrated that the knockdown of TRIM8 via the CRISPR/Cas9 gene editing technique significantly diminished the proliferative capacity and augmented apoptosis in Ewing sarcoma cells. This research, for the first time, identified TRIM8 as a regulator of EWSR1-FLI1, facilitating the survival of Ewing sarcoma cells by enhancing the stability and transcriptional activity of EWSR1-FLI1. Currently, chemotherapy is a crucial component of the treatment of Ewing sarcoma and usually includes the use of drugs such as cisplatin, cyclophosphamide, and etoposide. Additionally, targeted therapies for Ewing sarcoma have emerged in recent years.^70^, ^71^, ^72^ Mo et al^73^ identified the FACT (facilitates chromatin transcription) complex as a potential therapeutic target for Ewing sarcoma by integrating dataset analysis and drug screening. The therapeutic efficacy of CBL0137, a FACT-targeting drug, on tumors was validated *in vitro* and *in vivo* Ewing sarcoma models. The results showed that the FACT inhibitor drug significantly inhibited the growth of tumor cells. Mechanistically, CBL0137 significantly reduced mitotic activity and induced tumor cell death by targeting the EWSR1-FLI1-FACT pathway and disrupting the transcription of EWSR1-FLI1, SSRP1 (structure-specific recognition protein 1), and their downstream effectors. These results suggest that inhibiting the FACT complex reduces EWSR1-FLI1 activity, thereby affecting the growth and survival of tumor cells.

Rhabdomyosarcoma is a rare soft tissue malignancy that occurs primarily in children and adolescents, and it can appear in various parts of the body, including the eyes, nose, throat, genital tract, and limbs. Histologically, rhabdomyosarcomas are mainly classified into alveolar and embryonal types. Alveolar-type rhabdomyosarcoma is characterized by the presence of PAX-FOXO1 fusion genes, particularly PAX3-FOXO1 and PAX7-FOXO1. These fusion genes are formed by translocations to chromosomes t(2; 13) (q35; q14) and t(1; 13) (p36; q14), respectively.^74^^,^^75^ Missiaglia et al^76^ conducted an extensive analysis of clinical and molecular biology data from a substantial cohort of patients, revealing that individuals positive for the PAX3-FOXO1 fusion gene generally exhibit a poorer prognosis. These patients also show varied survival rates and treatment responses across different risk groups. The study identified the PAX3-FOXO1 fusion gene as the principal etiological factor in alveolar rhabdomyosarcoma. The researchers discovered that the fusion protein activates the target via transcription and contributes to the progression of rhabdomyosarcoma by stimulating cell proliferation, enhancing cell survival, inhibiting terminal differentiation, and influencing angiogenesis. Based on these findings, the team proposed an innovative risk stratification strategy centered on the fusion gene status. This approach is anticipated to improve the accuracy of prognostication in rhabdomyosarcoma patients, thereby aiding in the development of more targeted and personalized treatment strategies. Milewski et al^77^ found that PAX3-FOXO1 fusion protein is a key oncogenic driver of rhabdomyosarcoma, and PAX3-FOXO1 knockdown reduced FOXF1 mRNA and protein expression in RH4 and RH18 FP-RMS cells, which in turn regulates the development of rhabdomyosarcoma.

The ETV6-NTRK3 fusion gene is identified in various malignant tumors, with congenital fibrosarcoma being one of the earliest and most frequently associated tumor types.^78^ Congenital fibrosarcoma generally presents during infancy and is among the most common soft tissue neoplasms in pediatric patients. This tumor is characterized by a favorable prognosis, attributed to its relatively low malignant potential and limited propensity for metastatic dissemination. The ETV6-NTRK3 fusion gene arises from a chromosomal translocation (t(12; 15) (p13; q25)) involving the juxtaposition of the ETV6 gene located on chromosome 12 and the NTRK3 gene on chromosome 15. This fusion gene encodes a chimeric protein characterized by aberrant functional activity. Specifically, the fusion protein comprises the DNA-binding domain of ETV6 and the tyrosine kinase domain of NTRK3. This structural configuration confers constitutive tyrosine kinase activity upon the protein, thereby facilitating the autonomous activation of various downstream signaling pathways that drive cellular proliferation.^79^^,^^80^ Park et al^81^ elucidated the pivotal function of STAT1 in tumorigenesis mediated by the ETV6-NTRK3 fusion gene. Utilizing cell lines harboring the ETV6-NTRK3 fusion gene alongside a murine model, the study investigated the modifications in signaling pathways within tumor cells. The findings revealed that the ETV6-NTRK3 fusion gene precipitated a substantial activation of STAT1, both directly and indirectly, thereby inducing aberrant expression of downstream genes and facilitating tumor cell survival and invasiveness. Furthermore, the proliferation of tumor cells was significantly reduced when STAT1 was either knocked down or inhibited, suggesting that the abnormal activation of STAT1 is a crucial factor in the pathogenesis of ETV6-NTRK3-positive tumors. The main therapeutic approaches for congenital fibrosarcoma include surgical resection and chemotherapy.^82^ A European study^83^ reported a 75% response rate to chemotherapy in patients with congenital fibrosarcoma. The overall survival rate for these patients was 89% at both five-year and ten-year intervals. In recent years, advancements in research and the widespread application of targeted therapies have highlighted the potential of targeting fusion genes as a promising treatment strategy. Notably, TRK (tropomyosin receptor kinase) inhibitors have demonstrated substantial efficacy, particularly in cases of recurrence or where surgical resection is challenging, thereby offering the potential to significantly improve patient survival rates.^84^^,^^85^ DuBois et al^86^ have investigated the efficacy of larotrectinib as a neoadjuvant therapy in pediatric patients presenting with locally advanced, surgically challenging tumors. The findings revealed that larotrectinib administration resulted in a significant reduction in tumor size in most cases, thereby enhancing the feasibility of subsequent surgical resection. Furthermore, some patients achieved pathological remission, suggesting a decrease in tumor cell activity following treatment with larotrectinib. This observation indicates that the drug demonstrates an enhanced biological inhibitory effect on TRK-positive sarcomas. The ETV6-NTRK3 fusion gene is also identified in a rare subtype of breast cancer known as secretory breast cancer. The expression of the fusion protein is a key driver of secretory breast cancer pathogenesis, whereas it is infrequently observed in other breast cancer subtypes. Therefore, the identification of the fusion gene in breast cancer serves as the definitive criterion for diagnosing secretory breast cancer. Consequently, the identification of the fusion gene in breast cancer serves as the gold standard for diagnosing secretory breast carcinoma. Notably, secretory breast cancer generally exhibits a more favorable prognosis and can be effectively treated with targeted therapies utilizing TRK and NTRK inhibitors, demonstrating comparable efficacy.^87^^,^^88^ However, additional research is necessary to address the challenge of drug resistance, thereby ensuring the ongoing optimization of patient outcomes and long-term prognoses.

## Roles of fusion genes in thyroid cancer

RET (rearranged during transfection)-PTC (papillary thyroid carcinoma) is a common fusion gene in thyroid cancer that has been associated with various factors such as radiation exposure, genetic susceptibility, autoimmune diseases, obesity, and abnormal iodine intake.^89^, ^90^, ^91^, ^92^ The RET-PTC fusion gene is a fusion of the RET gene and the PTC gene to produce a new gene. The RET gene is located on human chromosome 10 and encodes a receptor tyrosine kinase, which typically regulates cell growth and differentiation. The PTC gene, which also encodes a tyrosine kinase, has been found to be implicated in the development of thyroid cancer.^93^^,^^94^ There are various specific forms of RET-PTC fusion genes, such as RET-PTC1, RET-PTC2, and RET-PTC3, each of which may have different effects on cancer development and prognosis.^93^ The relationship between the RET-PTC fusion gene and the prognosis of thyroid cancer is becoming clearer. Su et al^95^ found that RET gene rearrangement was significantly correlated with tumor multifocality in a study of 114 patients with PTC. This was confirmed by Yip et al^96^ who analyzed 1510 thyroid cancer patients and found that patients with the RET-PTC fusion gene had the highest rate of distant metastasis. These findings suggest that the RET-PTC fusion gene may serve as a molecular marker of poor prognosis in thyroid cancer. RET acts as a receptor tyrosine kinase that regulates cell growth and differentiation through receptor-ligand binding and self-phosphorylation. However, when RET is fused with PTC, its activity can be activated even in the absence of a ligand, thereby activating the downstream signaling pathway that promotes cell proliferation and survival.^97^ This gene fusion may result in abnormal proliferation and progression of thyroid cells. Mechanistically, the RET-PTC fusion gene can promote the activation of the MAPK signaling pathway, thereby promoting aberrant cell proliferation.

Surgical resection is usually the treatment of choice for most patients with RET-PTC-positive thyroid cancer. In recent years, targeted therapeutic agents against RET gene fusion have been extensively studied and have been clinically applied. Some small-molecule targeted agents, such as sorafenib and vandetanib, have been shown to be therapeutic for RET-PTC-positive thyroid cancer patients.^98^^,^^99^ Alao et al^100^ investigated the potential of the SPP86 kinase inhibitor as a therapeutic agent for treating thyroid cancer, which selectively inhibited RET-mediated cell proliferation and had no significant effect on RET-unmutated cells.

## Roles of fusion genes in prostate cancer

The incidence of prostate cancer is the fourth highest among all tumors, with higher rates in Europe and the United States and lower rates in East and South Asia.^18^ Prostate cancer may not have any noticeable symptoms in its early stages. As the tumor grows, a common symptom in advanced stages is blood in the urine and semen. In 2005, researchers identified the TMPRSS2 (transmembrane serine protease 2)-ERG (ETS-related gene) fusion gene in prostate cancer for the first time. TMPRSS2 and ERG are two different genes located on chromosome 21. Due to chromosomal rearrangements, the promoter region of TMPRSS2 is fused to the coding region of ERG. Studies have shown that the TMPRSS2-ERG fusion gene has a prevalence of up to 50% in prostate cancer. This fusion gene is highly specific, making it a specific marker for prostate cancer. The pathogenicity of the TMPRSS2-ERG fusion gene is mainly related to its ability to cause dysregulation of the ERG gene expression. The TMPRSS2 gene fragments contain androgen receptor binding sites, which are typically regulated by androgens. In the fusion gene, the TMPRSS2 gene fragment causes the overexpression of the ERG gene, a transcription factor that regulates gene expression, and its overexpression results in the abnormal activation of genes that regulate the cell cycle, promotes cell proliferation, and interferes with cell differentiation, thereby promoting tumorigenesis and metastasis.^101^^,^^102^ Mounir et al^103^ investigated the oncogenic mechanism of the TMPRSS2-ERG fusion gene. They found that TMPRSS2-ERG could inhibit neuroendocrine and tubulointerstitial cell differentiation, thereby promoting prostate cancer proliferation.

Song et al^104^ performed a meta-analysis and found that the TMPRSS2-ERG fusion gene occurred predominantly in patients with advanced T-stage, distant metastases, Gleason score ≤7, age ≤65 years, and high prostate-specific antigen levels (>10 ng/mL). Holly et al^105^ observed that obesity impacted the prognosis of patients with TMPRSS2-ERG fusion gene-positive prostate cancer. Therefore, they investigated the relationship between the TMPRSS2-ERG fusion gene and related factors such as insulin and hyperglycemia. High glucose levels were found to increase the abundance of IGFBP-2 (insulin-like growth factor binding protein 2), leading to an increase in the TMPRSS2-ERG fusion gene. The primary treatments for prostate cancer remain surgery, radiotherapy, and immunotherapy. Prostate cancer cell growth is dependent on androgens, so specific androgen deprivation therapies have been used to slow or inhibit their growth. Currently, there are no approved drugs that directly target the TMPRSS2-ERG fusion gene. It is hoped that future research will focus on developing small-molecule drugs that target TMPRSS2-ERG or its downstream pathway.

## Conclusion

The significance of fusion genes in tumor biology and clinical practice has been widely recognized. However, there are still many unexplored areas that require further investigation. Firstly, it is necessary to gain a deeper comprehension of the formation and mechanism of action of fusion genes, particularly the expression patterns and functional regulation mechanism in different types of tumors. Through an in-depth study of the functions and pathogenic mechanisms of fusion genes, we can discover new therapeutic targets and develop more effective strategies to improve the survival rate of tumor patients. Secondly, exploring the potential application of fusion genes in tumor diagnosis and prognosis assessment will help to establish a more accurate tumor typing system and prognosis assessment model, which will provide a reliable basis for clinical decision-making. Additionally, fusion genes can be used as predictive biomarkers for targeted therapies, helping to select the most appropriate therapeutic regimens and achieve personalized treatment. Finally, studying the interaction between fusion genes and tumors needs to be strengthened, particularly their interaction with the tumor microenvironment and the immune system. Understanding the regulatory networks and signaling pathways of fusion genes in tumorigenesis and development will help reveal the mechanisms of drug resistance and metastasis of tumors, and provide theoretical support for developing new immunotherapeutic strategies and combination therapeutic regimens. In conclusion, the relationship between fusion genes and tumors is complex. Future research may reveal more associations between fusion genes and tumors, thus providing more effective strategies for preventing, diagnosing, and treating tumors. This could make significant contributions to human health and well-being.

## Funding

This work was supported in part by grants from the 10.13039/501100001809National Natural Science Foundation of China (No. 82302987, 82303534, 82203233, 82202966, 82173142), the 10.13039/501100004735Natural Science Foundation of Hunan Province, China (No. 2024JJ4025, 2023JJ60469, 2023JJ40413, 2023JJ40417, 2023JJ30372, 2023JJ30375), the Science and Technology Innovation Program of Hunan Province, China (No. 2024RC3231, 2023ZJ1122, 2023RC3199, 2023SK4034, 2023RC1073), the Research Project of Health Commission of Hunan Province, China (No. R2023040, Z2023086, R2023093, 202203034978, 202202055318), and the Hunan Cancer Hospital Climb Plan (Hunan, China) (No. ZX2020001-3, YF2020002, 2023NSFC-A001, 2023NSFC-A002, 2023NSFC-A004).

## CRediT authorship contribution statement

**Haiqiong Tang:** Writing – original draft, Data curation. **Qiu Peng:** Writing – review & editing, Investigation. **Linda Oyang:** Investigation, Data curation. **Shiming Tan:** Data curation. **Xianjie Jiang:** Investigation. **Zongyao Ren:** Data curation. **Xuemeng Xu:** Data curation. **Mengzhou Shen:** Data curation. **Haofan Li:** Data curation. **Mingjing Peng:** Data curation. **Longzheng Xia:** Data curation. **Wenjuan Yang:** Data curation. **Shizhen Li:** Data curation. **Jiewen Wang:** Data curation. **Yaqian Han:** Methodology. **Nayiyuan Wu:** Project administration. **Yanyan Tang:** Data curation. **Jinguan Lin:** Investigation. **Qianjin Liao:** Supervision, Funding acquisition, Conceptualization. **Yujuan Zhou:** Supervision, Conceptualization.

## Conflict of interests

The authors declared no competing interests.
